# A short peptide derived from pigment epithelial-derived factor exhibits an angioinhibitory effect

**DOI:** 10.1186/s12886-022-02295-0

**Published:** 2022-02-22

**Authors:** Tsung-Chuan Ho, Shu-I Yeh, Show-Li Chen, Ting-Wen Chu, Yeou-Ping Tsao

**Affiliations:** 1 Department of Medical Research, Mackay Memorial Hospital, No. 45, Minsheng Rd., Tamsui District, New Taipei City, 25160 Taiwan; 2 Department of Medicine, Mackay Medical College, Zhongzheng Rd., Sanzhi Dist, New Taipei City, 25245 Taiwan; 3 Department of Ophthalmology, Mackay Memorial Hospital, No. 92, Sec. 2, Chung Shan North Road, Taipei, 10449 Taiwan; 4 Graduate Institute of Microbiology, College of Medicine, National Taiwan University, 7F, No. 1, Sec. 1, Jen-Ai Rd., Taipei, 10617 Taiwan

**Keywords:** Pigment epithelial-derived factor, Vascular endothelial growth factor, Peptide, Corneal neovascularization, Apoptosis

## Abstract

**Background:**

Pigment epithelial-derived factor (PEDF), a 50 kDa secreted glycoprotein, exhibits distinct effects on a range of cell types. PEDF has been shown to inhibit vascular endothelial growth factor (VEGF)-mediated angiogenesis and widely accepted as a promising agent for treatment eye diseases related to neovascularization. A pool of short peptide fragments derived from PEDF reportedly manifests angioinhibitory activity. This study aims to determine the minimal PEDF fragment which can exert the anti-VEGF effect.

**Methods:**

A series of shorter synthetic peptides, derived from the 34-mer (PEDF amino acid positions Asp44-Asn77), were synthesized. An MTT assay was used to evaluate the ability of the 34-mer-derived peptides to inhibit VEGF-induced proliferation of multiple myeloma RPMI8226 cells. Cell apoptosis was monitored by annexin V-FITC staining. Western blot analysis was used to detect phosphorylated kinases, including c-Jun N-terminal kinase (JNK) and p38 mitogen-activated protein kinase (MAPK), and the expression of apoptosis-associated proteins, including p53, bax and caspase-3. VEGF-mediated angiogenesis of human umbilical vein endothelial cells (HUVECs), rat aortic ring and mouse cornea were used to detect the angioinhibitory activity of the PEDF-derived peptides.

**Results:**

The MTT assay showed that the anti-VEGF effect of a 7-mer (Asp64-Ser70) was 1.5-fold greater than the 34-mer. In addition, massive apoptosis (37%) was induced by 7-mer treatment. The 7-mer induced JNK phosphorylation in RPMI8226 cells. Cell apoptosis and apoptosis-associated proteins induced by the 7-mer were blocked by pharmacological inhibition of JNK, but not p38 MAPK. Moreover, the 7-mer prevented VEGF-mediated angiogenesis of endothelial cells (ECs), including tube formation, aortic EC spreading and corneal neovascularization in mice.

**Conclusions:**

This is the first study to show that the PEDF 7-mer peptide manifests anti-VEGF activity, further establishing its potential as an anti-angiogenic agent.

**Supplementary Information:**

The online version contains supplementary material available at 10.1186/s12886-022-02295-0.

## Background

Pigment epithelial-derived factor (PEDF) was initially identified in the culture supernatant of human fetal retinal pigment epithelial cells, with the ability to promote neuronal differentiation of retinoblastoma Y79 cells [1]. Further studies showed PEDF to be a potent angiogenic inhibitor and the pathological neovascularization in retina is associated with low levels of PEDF in the ocular vitreous humor [2, 3]. Presently, full-length PEDF is considered to be less suitable as an anti-angiogenic agent than PEDF-derived active fragments because of several issues, including antigenicity, stability, production cost and, perhaps, its multifunctional nature [4, 5]. In this regard, the 34-mer and a shorter peptide within the 34-mer (an 18-mer, Asn60-Asn77) were investigated for the anti-angiogenic activity of PEDF, including induction of endothelial cell apoptosis, blockage of VEGF-induced neovessel formation, analyzed by a mouse corneal pocket assay, and reduction of vascular density in tumor tissues [4, 6]. In addition, the 34-mer and 18-mer have been shown to have no neurotrophic activity, suggesting that they posses only PEDF anti-angiogenic function [4, 6, 7].

VEGF is an important proangiogenic factor responsible for the development and formation of blood vessels. VEGF binds to the tyrosine kinase receptors of vascular endothelial cells to induce cell proliferation, migration and sprouting angiogenesis [8]. In addition, VEGF is important for maintenance of the integrity of pericyte-endothelial interactions in the retinal microvasculature [9]. VEGF is strongly involved in several angiogenesis-driven eye diseases. For example, anti-VEGF drugs, including pegaptanib (Macugen) and ranibizumab (Lucentis), have been approved for treating retinal and choroidal vascular diseases, such as diabetic macular edema and exudative age-related macular degeneration (AMD). Notably, it has been found that many exudative AMD patients have a persistent hemorrhage and progressive fibrosis at the retinal fovea, following long-term treatment with current anti-VEGF drugs, rendering significant unmet medical needs [10]. VEGF also has a particular angiogenesis-independent effect. For example, VEGF can promote proliferation of multiple myeloma cells in culture [11, 12].

Development of PEDF-based angioinhibitory therapies may be helpful for resolving the problem of persistent side effects associated with current anti-VEGF drugs. In this study, a series of shorter peptides covering the 34-mer were synthesized. We investigated their anti-VEGF activity using an established model of multiple myeloma RPMI8226 cell growth induced by VEGF. The 7-mer had effective anti-VEGF activity. Moreover, we demonstrated for the first time that the 7-mer was able to abolish VEGF-induced angiogenesis in vitro and in a mouse model of corneal neovascularization.

## Methods

### Materials

**Fig. 1:**
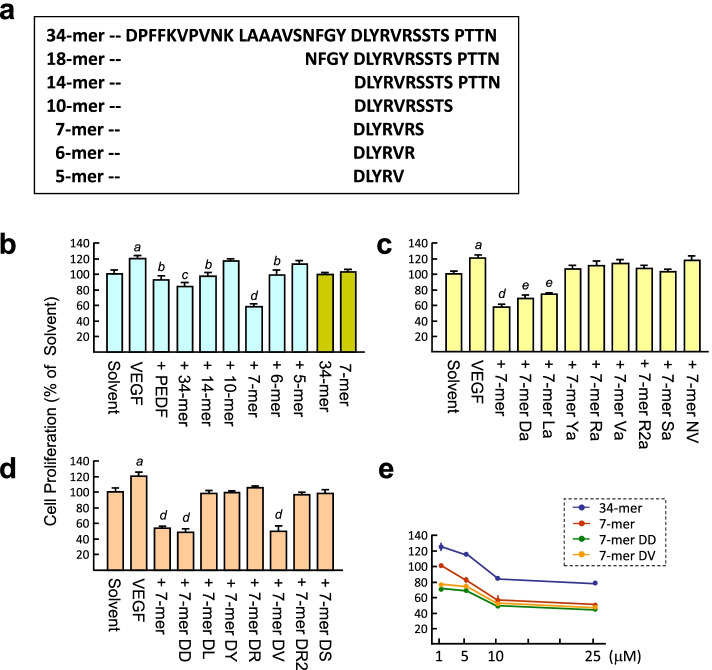
Analysis of the effect of 34-mer derived peptides on the VEGF-induced RPMI8226 cell proliferation. **a** Sequence diagram of the peptides derived from the 34-mer. **b-e** VEGF-induced cell proliferation was measured by an MTT assay. Cells were treated with 10 ng/ml VEGF in the presence of 25 μM peptide for 24 h. The MTT value of peptide solvent-treated RPMI8226 cells was set at 100%. Results represent the mean ± SD from three independent experiments. ^*a*^*P* < 0.05 versus solvent. ^*b*^*P* < 0.001, ^*c*^*P* < 0.0001, ^*d*^*P* < 0.00002 and ^*e*^*P* < 0.00004 versus VEGF treatment

VEGF and TACS annexin V-FITC kits were purchased from R&D Systems (Minneapolis, MN, USA). Matrigel was purchased from BD Biosciences (New Bedford, MA, USA). HUVECs were purchased from Cascade Biologics, Inc. (Portland, OR, USA). Hydron (529265), sucrose octasulfate–aluminum complex (S0652) were from Sigma-Aldrich (St. Louis, MO, USA). Culture medium, low-serum growth supplement (LSGS) and fetal bovine serum (FBS) were from Gibco-BRL. 3-(4,5-cimethylthiazol-2-yl)-2,5-diphenyl tetrazolium bromide (MTT) was from Merck (Catalog number 1.11714.0001). SB203580 (p38 MAPK inhibitor) and SP600125 (JNK inhibitor) were purchased from SelleckChem (Houston, TX, USA). All peptides shown in Fig. 1a were synthesized by GenScript (Piscataway, NJ, USA), each peptide being modified by acetylation at the NH_2_ terminus and amidation at the COOH terminus to improve its stability, with subsequent characterization by mass spectrometry (> 95% purity). Peptides were reconstituted in DMSO as stock (10 mM).

### Culture of RPMI8226 cells and MTT assay

Human multiple myeloma cells, RPMI8226 were purchased from Bioresource Collection and Research Center (Hsinchu, Taiwan). Cells were cultivated in RPMI1640 medium supplemented with 10% FBS, 50 μg/ml penicillin, 50 μg/ml streptomycin, and 2 mM L-glutamine at 37 °C in a 5% CO_2_ atmosphere. Cell viability was determined by an MTT assay. Cells were cultured in serum-free medium for 24 h, and then seeded in 48-well culture plates (3 × 10^5^ cells/well) and cultured in 0.5 ml fresh serum-free RPMI1640 medium supplemented with 10 ng/ml VEGF and 20 μM PEDF peptide for a further 24 h. Subsequently, 50 μl of the MTT stock solution (5 mg MTT dissolved in 1 ml of sterile PBS) was added each well, and incubated at 37 °C for 4 h. MTT stock solution added to medium alone was included as a negative control. Aliquots (450 μl) from each sample were transferred to a new well of a 48-well culture plate, 100 μl DMSO added with thorough mixing, and allowed to react at 37 °C for 20 min before reading the absorbance at 570 nm.

### Cell culture of HUVECs

Cells were grown in Medium 200 with LSGS (supplement contains 1.9% fetal bovine serum, 3 ng/ml bFGF, 10 μg/ml heparin, 1 μg/ml hydrocortisone, and 10 ng/ml EGF). Culture plates were coated with 2% gelatin. Cells (passages 4–8) were cultured at 37 °C in a humidified atmosphere of 5% CO2. For peptide treatment, HUVECs were cultured in low serum (0.5% FBS) medium containing 20 ng/ml VEGF and 7-mer peptide (20 μM, unless otherwise specified).

### Endothelial cell capillary-like tube formation assay

Growth factor-reduced matrigel was pipetted into pre-chilled 24-well plates (150 μl matrigel per well) and polymerized for 45 min at 37 °C. HUVECs (4 × 10^4^ per well) in low serum (0.5% FBS) medium were treated with 20 ng/ml VEGF and 7-mer (10 μM) in matrigel coated plates. After 6 h of incubation, tubular structures were photographed. Images were captured using a Cannon camera on a Zeiss inverted microscope with magnification × 40.

### Western blot analysis

Cell lysis and SDS–PAGE were as described previously [13]. Antibodies used in this study were for active p38 MAPK and active JNK (Promega, Madison, WI), p38 MAPK/SAPK2, JNK (Upstate Biotechnology, Lake Placid, NY), p53 (Chemicon, Temecula, CA), bax, p21, cleaved caspase-3 and β-actin (Abcam Ltd., Cambridge, UK). Proteins of interest were detected using the appropriate IgG-horseradish peroxidase-linked secondary antibody (Santa Cruz Biotechnology) and enhanced chemiluminescence reagent (Amersham, Arlington Heights, IL). X-ray films were scanned on a model GS-700 Imaging Densitometer (Bio-Rad, Hercules, CA) and analyzed with Labworks 4.0 software. For quantification, blots from at least three independent experiments were used.

### Annexin-V/propidium iodide (PI) double-staining assay

Cell apoptosis was examined using a TACS annexin V-FITC kit according to the manufacturer’s instructions for in situ staining. The cell numbers were monitored by counterstaining with Hoechst 33342. Afterward, apoptosis was examined and photographed by a Zeiss epifluorescence microscope (× 100, 10 fields/sample). Images were recorded on Zeiss software.

### Animal studies

All animals were housed in an animal room under temperature control (24–25 °C) and a 12:12 light-dark cycle. Standard laboratory chow and tap water were available ad libitum. Experimental procedures were approved by the Mackay Memorial Hospital Review Board (project code: MMH-A-S-103-10; New Taipei City, Taiwan). Animals in ophthalmic research were performed in compliance with national animal welfare regulations (Council of Agriculture, Taiwan, ROC), the ARVO Statement and the ARRIVE Guidelines. C57BL/6 mice with an age of 6 weeks were anaesthetized by an intraperitoneal injection of a mixture of ketamine (100 mg/kg body weight) and xylazine (10 mg/kg). All animals were euthanized by CO_2_ inhalation prior to dissection.

### Rat aortic ring assay

Thoracic aortas were removed from euthanized 10-week-old male Sprague Dawley rats and gently stripped of periaortic fibroadipose tissue. Aortas were sectioned into ~ 1 mm length rings, being manipulated in Medium 200. Matrigel (120 μl) mixed with 24 μl LSGS, 72 μl medium, 200 ng/ml VEGF and 100 μM 7-mer was used to embed the aortic ring and then polymerized in 12-well plates (37 °C for 30 min). Subsequently, 1 ml culture medium supplemented with 100 units/ml penicillin, and 100 ng/ml streptomycin was added. The cultures were propagated at 37 °C in a humidified incubator for up to 5 days. Sprouts were recorded using an inverted microscope platform (Leica DMI6000B) with bright-field optics. Each ring was scored by three independent observers on a scale 0–3, depending on the degree of vessel sprouting observed (0 = no sprouting, 3 = profuse sprouting).

### Mouse corneal micropocket assay

The assay was performed according to procedures described previously [14]. Briefly, corneal micropockets were created in 6-week-old C57BL/6 mice with the needle of 1-ml injector. A micropellet of sucrose aluminum sulfate and hydron polymer containing 200 ng VEGF, with or without 2.5 μg 7-mer peptide, was delivered into each corneal pocket. Eyes were photographed by a slit-lamp biomicroscope on day 7 after micropellet implantation. Ingrowth of the blood vessels into the avascular cornea, towards the pellet, was scored as a positive response. The results are reported as the fraction of positive corneas of the total implanted.

### Statistics

The data were generated from at least three independent experiments. All numerical values are expressed as the mean ± SD. Comparisons of two groups were made using the Mann–Whitney test. *P* < 0.05 was considered significant.

## Results

### Identification of an active core region in the PEDF 34-mer, in terms of its anti-VEGF activity

We used an MTT assay to measure the growth and survival of multiple myeloma RPMI8226 cells induced by VEGF. PEDF blocked the VEGF effect (93 ± 3.8% versus 120 ± 3.9%; untreated cells set as 100%; Fig. 1b), similar to previous reports [11, 15] and the 34-mer displayed a stronger inhibitory effect than PEDF (84 ± 4.0%). The 14-mer, missing the 20 amino acids at the N-terminal end of the 34-mer, had a weaker anti-VEGF effect (97 ± 3.6%). We deleted the amino acid residues at the C-terminal end of the 14-mer to generate a 10-mer, 7-mer, 6-mer, and 5-mer and these yielded values of 116 ± 2.64%, 58 ± 4.1%, 99 ± 6.2% and 113 ± 4.2%, respectively, in the MTT assay. The 10-mer and 5-mer failed to suppress the VEGF effect, but the 6-mer had the same anti-VEGF activity as the 14-mer. Unexpectedly, the 7-mer exhibited more potent anti-VEGF activity than the 34-mer (~ 1.5-fold). Notably, treatment of RPMI8226 cells with the 34-mer and 7-mer in the absence of VEGF showed that neither peptide had a significant cytotoxic effect. This result supports the previous finding that the anti-angiogenic activities of PEDF and the 34-mer are involved in selectively inducing apoptosis of proliferating ECs, rather than quiescent ECs [16]. In addition, as depicted in Fig. 1c, single alanine substitutions of the Y66, R67, V68, R69 and S70 residues of the 7-mer, cause a large drop in the anti-VEGF activity of the 7-mer (97.1% ~ 106.9% versus 57.3%), whereas substitutions of the D64 and L65 residues cause a modest decrease in anti-VEGF activity (69 ± 3.5% and 74 ± 0.9%). We prepared the D64N/L65V double mutant of the 7-mer to determine whether this peptide retained anti-VEGF activity. However, the D64N/L65V double mutation was deleterious to the anti-VEGF activity of the 7-mer (117 ± 4.1%). Collectively, truncation of the 34-mer peptide identifies the 7-mer as a core active region of the 34-mer, in terms of its anti-VEGF effect on RPMI8226 cells. The results also reveal that the anti-VEGF activities of 34-mer-derived peptides are varied and associated somewhat with amino acid residues near the N- and C-termini of the 7-mer region.

### The D-form 7-mer peptide exerts higher anti-VEGF effect in vitro than its L-form isomer

Natural L-amino acids are unstable in vivo because they are susceptible to protease degradation. To improve the resistance of the 7-mer to proteolysis, D-amino acid substitution was used for peptide modification. Using D-amino acids for peptide modification have been suggested potentially to promote the peptide therapeutic activity [17]. To investigate whether 7-mer modified by D-amino acids can enhance its anti-VEGF activity in the RPMI8226 cells, an MTT assay revealed that replacement of the D64 and V68 residues of the 7-mer by D-amino acids (7-mer DD and 7-mer DV) retained anti-VEGF activity comparable to the 7-mer (49 ± 4.3% and 49 ± 7.0%, 54 ± 1.5%). whereas On the other hand, D-amino acid substitution of the other residues of the 7-mer yielded peptides with much less activity against the VEGF effect on RPMI8226 cells (~ 105–98%) at the tested concentration (25 μM; Fig. 1d). Next, the VEGF-treated RPMI8226 cells were exposed to various doses of the 7-mer peptide (1, 5, 10 and 25 μM). An MTT assay showed that treatment with the 7-mer, 7-mer DD and 7-mer DV decreased cell viability in a dose-dependent manner (Fig. 1e). We noted that the 7-mer DD and 7-mer DV had a greater anti-VEGF effect than the 7-mer at the lowest concentration (1 μM; 75 ± 2.8% and 78 ± 1.7% versus 101 ± 1.2%). In contrast, cell viability decreased significantly with the 34-mer treatment at 10 and 25 μM, but not at 1 and 5 μM. Taken together, these results indicate that the D-form 7-mer peptide has a greater anti-VEGF activity than its L-form.

### The 34-mer and 7-mer induce RPMI8226 cell apoptosis

**Fig. 2:**
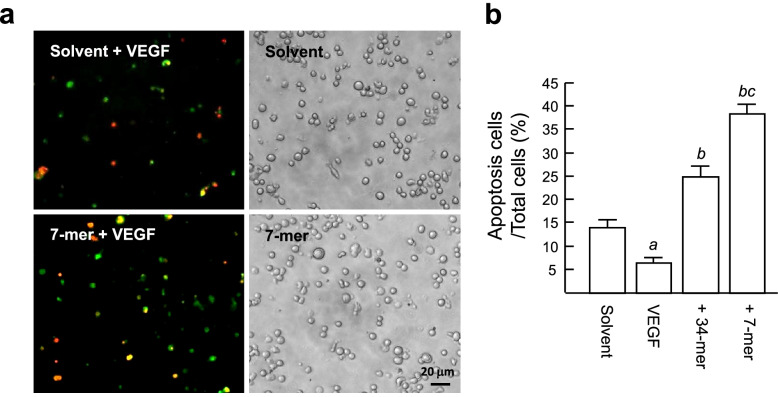
The 34-mer and 7-mer induce apoptosis of RPMI8226 cells. **a** Fluorescence microscopy of annexin V-FITC/PI double staining. Treatment of RPMI8226 cells was as described in Fig. 1. Apoptosis was determined by annexin V-FITC staining (green dots) and double stained with propidium iodide (red dots). A representative of three independent experiments is shown. **b** The percentage of apoptotic cells was quantified by dividing the number of annexin V-positive cells by the total number of cells counted by phase-contrast light microscopy. Control peptide indicates cells were treated with the 5-mer. Histograms show means ± SD. ^*a*^*P* < 0.005 versus peptide solvent-treated cells. ^*b*^*P* < 0.0002 versus VEGF-treated cells. ^*c*^*P* < 0.001 versus 34-mer-treated cells

We next investigated whether the 34-mer and 7-mer can induce apoptosis of VEGF-treated RPMI8226 cells by annexin V-FITC/PI double staining. This technique has been used to detect viable cells (annexin V^−^/PI^−^), cells undergoing early apoptosis (annexin V^+^/PI^−^), late apoptotic cells (annexin V^+^/PI^+^) and necrotic cells (annexin V^−^/PI^+^; Fig. 2a). The simultaneous detection of early and late apoptosis (annexin V^+^ cells) revealed that withdrawal of serum from the culture medium caused apoptosis of RPMI82226 cells, whereas VEGF could prevent this serum deprivation-induced apoptosis (annexin V^+^ cells: 14.0 ± 1.7% versus 6.5 ± 1.1%; Fig. 2b). Importantly, there was a significant increase of cell apoptosis after treatment with the 34-mer and 7-mer for 24 h (25.1 ± 2.2% and 38.6 ± 2.1%, respectively). In addition, Necrosis (annexin V^−^/PI^+^) was rare after cells received these treatments. In addition, we noted that part of apoptotic RPMI8226 cells displayed morphologic changes, including shrinkage and membrane blebbing, resulting in different sizes of green dots. A previous report also indicates that PPARγ overexpression in RPMI8226 cells induces cells undergoing apoptosis with morphologic hallmarks, such as shrinkage and membrane blebbing [18]. Taken together, these findings provide evidence that the 34-mer and 7-mer are able to suppress the VEGF-mediated cell protective effect by inducing cell apoptosis.

### The 7-mer triggers JNK-dependent apoptotic signaling in VEGF-treated RPMI8226 cells

**Fig. 3:**
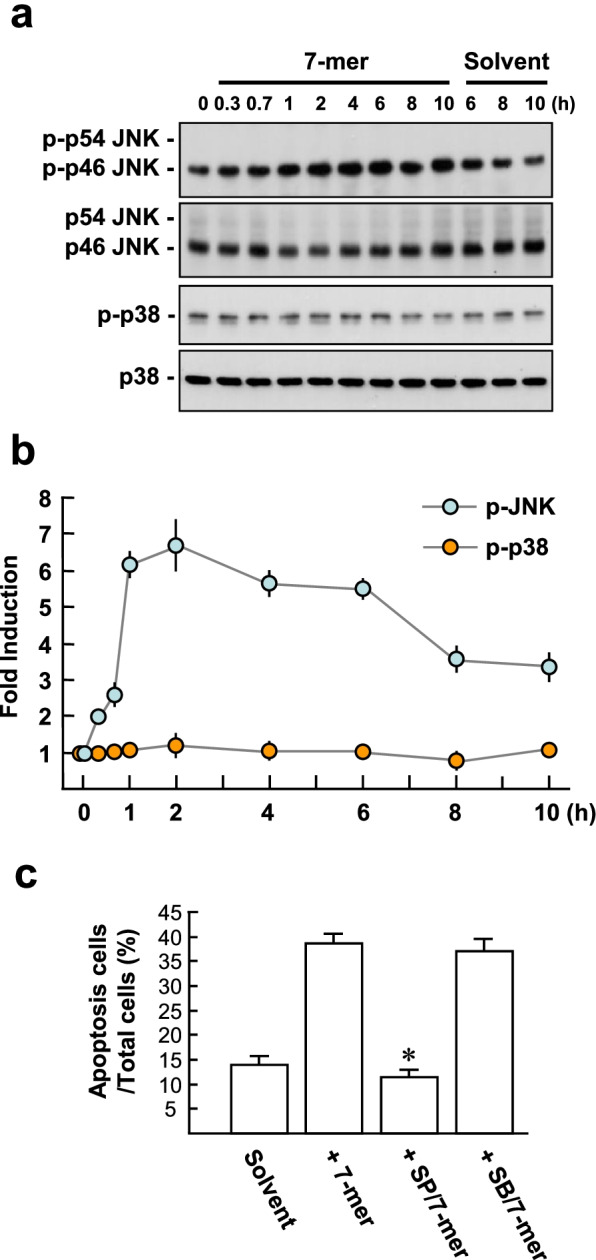
The 7-mer induces JNK signaling to trigger RPMI8226 cell apoptosis. 3 × 10^5^ cells were serum starved for 24 h and then exposed to 10 ng/ml VEGF and 25 μM 7-mer for the indicated time periods. **a** Western blotting was performed to detect the active phosphorylated forms of JNK (p-JNK) and p38 MAPK (p-p38), shown in the upper panels. Antibodies were then stripped and the blots were re-incubated with anti-JNK and anti-p38 MAPK antibodies (lower panels), respectively, to detect the levels of total JNK and p38 MAPK. **b** Densitometric analyses from three independent experiments are shown. **c** Cell were pretreated with 10 μM SP600125 or SB203580 for 10 min and then treated as described above for 24 h and apoptosis was quantified using the annexin V-FITC apoptosis detection kit. *P < 0.0001 versus 7-mer-treated cells

To investigate whether the 7-mer affects JNK and p38 MAPK signaling in RPMI82226 cells, western blot analysis was performed using antibodies against the active phosphorylated forms of JNK and p38 MAPK. An increase in p-p46 JNK was detected 20 min after 7-mer treatment, reaching a peak value at 2 h, and the phosphorylation was sustained for up to 10 h. Treatment with the 7-mer had no significant effect on p38 MAPK phosphorylation (Fig. 3a and b). In this regard, pretreatment of RPMI82226 cells with a JNK inhibitor (10 μM SP600125), but not in cells pretreated with a p38 MAPK inhibitor (10 μM SB203580), altered 7-mer-induced RPMI8226 cell apoptosis from 38.6 ± 2.1% to 11.6 ± 1.5%, assayed by annexin V-FITC staining (Fig. 3c).

**Fig. 4:**
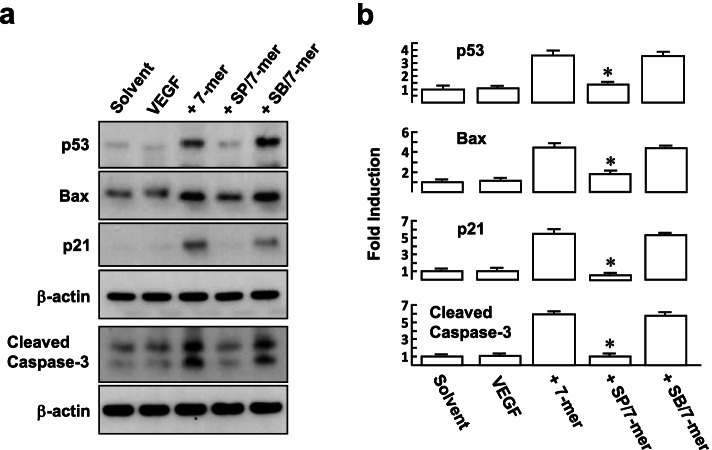
The 7-mer induces JNK signaling to trigger p53/bax/p21^Waf1^ expression in RPMI8226 cells. **a** and **b** Cell treatments were as described in Fig. 3. Cell lysates were then prepared for western blot analysis of p53, Bax, and p21. Representative blots and densitometric analyses from three independent experiments are shown. ^*^P < 0.001 versus 7-mer-treated cells

Overexpression and activation of p53 play a critical role in PEDF-induced endothelial cell apoptosis [19]. As shown in Fig. 4a, western blot analysis showed that treatment with the 7-mer for 8 h caused an increase of the protein levels of p53 and its transcriptional target genes, including *Bax* (an apoptosis inducer) and *p21*^*Waf1*^ (a cyclin-dependent kinase inhibitor) compared to solvent control (3.6 ± 0.39, 4.5 ± 0.40 and 5.5 ± 0.43 –fold; Fig. 4b). Moreover, an increase in p53 expression corresponded to the elevation of cleaved/active caspase-3, a frequently activated death protease in the apoptosis pathway, was observed following 7-mer treatment for 24 h. We determined whether JNK activation induced by the 7-mer can regulate the level of p53 protein. Western blotting revealed that increases in the levels of induced p53, bax, p21, and cleaved caspase-3 were significantly inhibited in cells pretreated with SP600125, but not in cells pretreated with SB203580, suggesting p-JNK engagement in RPMI8226 cell apoptosis or cell cycle arrest induced by the 7-mer. Taken together, our results reveal that apoptosis and the p53/Bax/p21^Waf1^ pathway induced by the 7-mer were mediated by JNK activation.

### The 7-mer exhibits the angioinhibitory activity in HUVECs

**Fig. 5:**
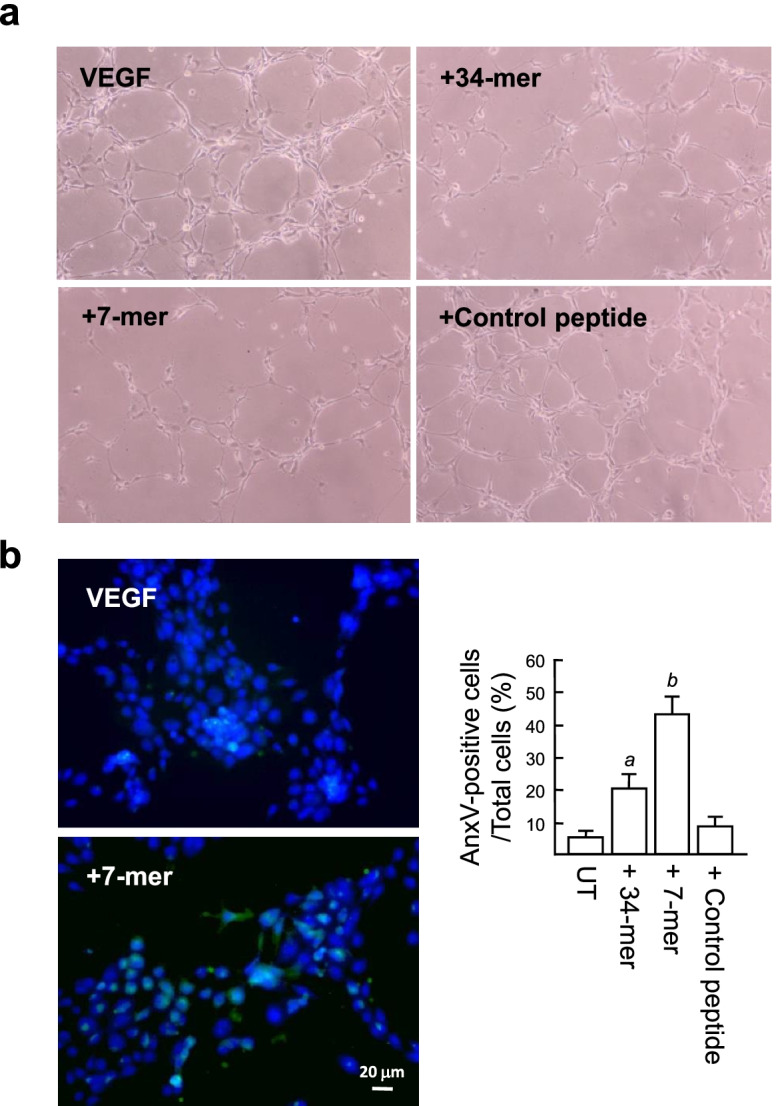
The 34-mer and 7-mer display angioinhibitory properties in culture. **a** VEGF-induced capillary-like tubular structures were observed in matrigel with phase-contrast light microscopy. The experiment was repeated three times. Magnification is × 40. **b** The 7-mer induces HUVEC apoptosis in the presence of VEGF. Cell apoptosis was analyzed using the Annexin V-FITC Apoptosis Detection kit according to the in situ staining protocol after treatment for 16 h. Double staining with an annexin V-FITC and the fluorescent dye Hoechst 33342 was used to visualize the apoptotic cells (green) and nuclei (blue), respectively. A representative result of four independent experiments is shown. The percentages of apoptotic cells were quantified. ^*a*^*P* < 0.005 versus VEGF-treated cells

ECs are converted to capillary tube-like structures in response to VEGF. We observed the VEGF effect on HUVECs using a matrigel-based tube formation assay. As depicted in Fig. 5a, exposure of HUVECs to VEGF for 6 h formed a massive tube-like network as described previously [20] (Fig. 5a). Treatment with the 7-mer and 34-mer resulted in a marked decrease of the VEGF-stimulated tube-like network. Next, we investigated whether the 7-mer can induce apoptosis of HUVECs. The annexin V-FITC staining showed that treatment of HUVECs with the 34-mer and 7-mer for 16 h increased the percentage of apoptotic cells from 6.3 ± 1.6% (VEGF-treated) to 20.1 ± 4.4% and 43.0 ± 5.5%, respectively (Fig. 5b). A control peptide (the 5-mer) had no such effect. These results indicate that the 7-mer has anti-angiogenic activity through arresting VEGF-mediated capillary morphogenesis and inducing endothelial cell apoptosis.

### The 7-mer inhibits VEGF-induced microvessel sprouting ex vivo and corneal neovascularization

**Fig. 6:**
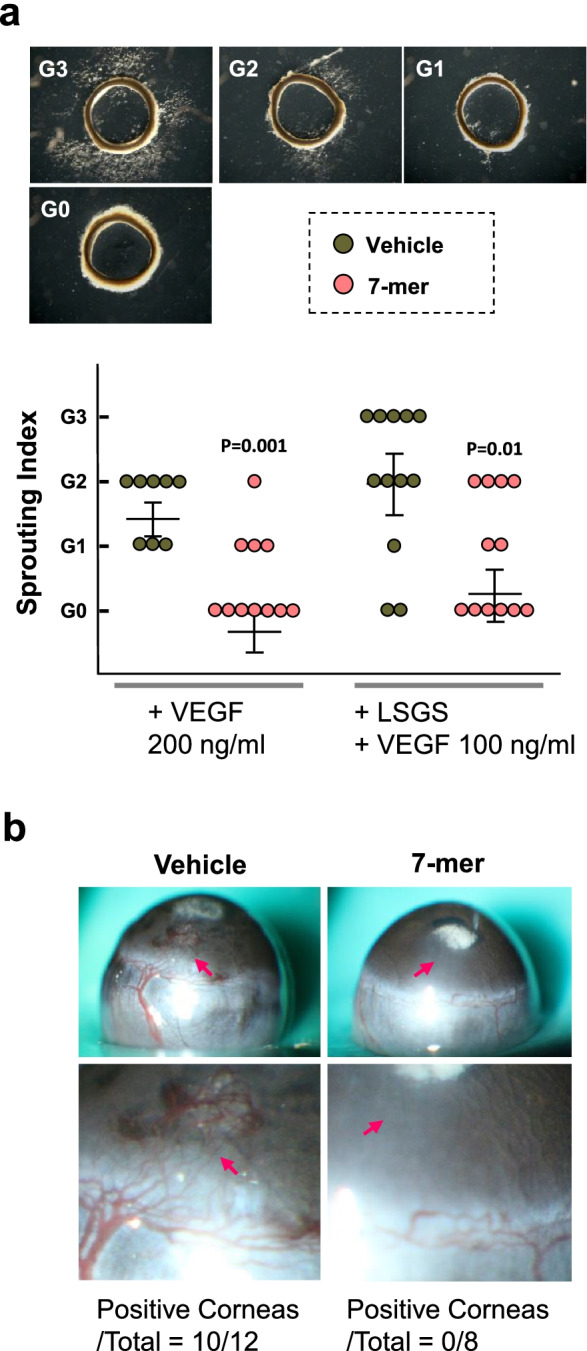
The effect of the 7-mer on VEGF-induced microvessel sprouting of aortic ring and corneal neovascularization. **a** Rat aortic ring assay. Photomicrographs of aortic ring explants after treatment for 5 days (magnification × 40). Sprouting was evaluated on a scale from 0 (no sprouts) to 3 (profuse sprouting). There was no sprouting of vessels from ex vivo aortic rings embedded in Matrigel without LSGS or VEGF. **b** The 7-mer inhibits VEGF-induced corneal angiogenesis in C57BL/6 mice. Representative corneas and final scoring determining the number of positive corneas per total implantations. Arrows indicate the corneal region near to the VEGF/vehicle or VEGF/7-mer pellet

The effect of the 7-mer on VEGF-induced microvessel sprouting was evaluated using a rat aortic ring assay. As shown in Fig. 6a, outgrowth of sprouts from the aortic rings was observed after VEGF treatment for 5 days, whereas this outgrowth was suppressed when the VEGF-treated aortic rings were exposed to the 7-mer (*P* = 0.001). In addition, aortic rings exposed to multiple angiogenic factors (LSGS and VEGF) produced profuse sprouts around the ring that were blocked by the 7-mer. Furthermore, in a mouse corneal model of neovascularization, VEGF-induced neovessels in the corneas of C57BL/6 mice were prevented by the 7-mer (Fig. 6b). The results imply that the 7-mer is active against angiogenesis in vivo.

.

## Discussion

Identification of angioinhibitory peptides derived from PEDF may be a promising strategy for improving the treatment of angiogenesis-related diseases. Previous studies have identified the 34-mer (D44-N77) and 18-mer (N60-N77) that preserve the anti-angiogenic and antitumor activities of human PEDF, but the 23-mer (A55-N77) and 14-mer (D64-N77) are devoid of PEDF activity [4]. For example, the 14-mer is unable to induce EC apoptosis in vitro. Here, we found for the first time that the 7-mer peptide (D64-S70), representing a minimal region within PEDF, reproduced the anti-VEGF effect, comparable to the 34-mer. Also, the longer peptides (34-mer, 14-mer and 10-mer) with N- and C- terminal sequences near the 7-mer region, displayed a rather poor inhibitory effect on VEGF, compared to the 7-mer peptide.

It has been reported that the vitreous humors of exudative AMD patients contain lower levels of PEDF [3]. This reduction of PEDF levels leads to patients’ vitreous insufficiency in suppressing EC migration, compared to vitreous humor from age-matched control donors [3]. These findings suggest that loss of PEDF may create a permissive environment for the pathogenesis of exudative AMD. Overexpression of PEDF in mice by intravitreous injection of an adenoviral vector encoding PEDF (AdPEDF) and PEDF transgenic mice have been reported to block laser-induced choroidal neovascularization (CNV) and VEGF-induced retinal neovascularization [21, 22]. This suggests that elevated levels of intraocular PEDF are beneficial in suppressing ocular neovascularization. However, treatment of ocular neovascular diseases with the wild-type (WT)-PEDF may generate additional unexpected effects through its multifunctional activities. These functional effects could come from the interaction of different PEDF domains with an individual receptor on the surface of different types of cells. PEDF is known that contributes to antiangiogenesis, antitumor, antiinflammation, neuroprotection and tissue repair by stem cells [23]. In addition, For example, in comparison with WT-PEDF, it has been found that mutation of putative phosphorylation sites in PEDF (phosphomimetic mutants) leads to more profound anti-angiogenic activity and may avoid the induction of tumor growth [24]. In addition, recombinant PEDF may create complex antigenicity [4]. In this regard, it has been proposed that synthetic anti-angiogenic peptides derived from PEDF are more practical as agents for treating neovascularization in tumors and the eye [4, 5].

In the present study, the 7-mer induced massive apoptosis of RPMI8226 cells and it is plausible to propose apoptosis as the major anti-VEGF mechanism of the 7-mer in culture, rather than cell cycle arrest alone. In addition, we found that the 7-mer was able to activate JNK signaling in RPMI8226 cells. Pharmacological inhibition of JNK with SP600125 suppressed the 7-mer induced RPMI8226 cell apoptosis. Meanwhile, SP600125 also suppressed the effect of the 7-mer on induction of p53, Bax, p21^waf1^ and active caspase-3 protein expression. Several studies have shown that the PEDF receptor (37/67 kDa laminin receptor; LR), JNK, PPARγ and p53 and effectors, including FasL and Bax, are associated with PEDF-induced EC apoptosis [16, 17, 25, 26]. In particular, it has been demonstrated that a neutralizing antibody against LR can block the effect of PEDF on VEGF-induced multiple myeloma cell proliferation [15]. These results suggest that PEDF-mediated apoptotic signaling acts on ECs and myeloma cells in a similar manner.

## Conclusions

In summary, identification of PEDF-derived angioinhibitory peptides may facilitate the development of more effective therapeutic strategies for angiogenesis-related eye diseases and potentially with less side effects. VEGF-activated myeloma cells and ECs are responsive to the 7-mer, providing evidence for the 7-mer as an anti-VEGF agent. Further preclinical animal studies of ocular neovascular diseases are required to evaluate the therapeutic efficacy of the 7-mer.

## Supplementary Information

**Additional file 1.**

## Data Availability

The datasets used and analysed during the current study are available from the corresponding author on reasonable request.

## Abbreviations

PEDF: Pigment epithelial-derived factor
VEGF: Vascular endothelial growth factor
JNK: c-Jun N-terminal kinase
MAPK: Mitogen-activated protein kinase
HUVECs: Human umbilical vein endothelial cells
ECs: Endothelial cells
AMD: Age-related macular degeneration
MTT: 3-(4,5-cimethylthiazol-2-yl)-2,5-diphenyl tetrazolium bromide
DMSO: Dimethyl sulfoxide
bFGF: Basic fibroblast growth factor
EGF: Epidermal growth factor
SDS–PAGE: Sodium dodecyl sulfate polyacrylamide gelelectrophoresis
PI: Propidium iodide
CNV: Choroidal neovascularization
