# Supplementary material for: A short peptide derived from pigment epithelial-derived factor exhibits an angioinhibitory effect
Source: BMC Ophthalmol. 2022 Feb 22;22:88. doi: 10.1186/s12886-022-02295-0 (PMC8864869; doi:10.1186/s12886-022-02295-0)
Supplement: Supplementary file 1 — Additional file 1. [file 12886_2022_2295_MOESM1_ESM.ppt]

## Slide 1
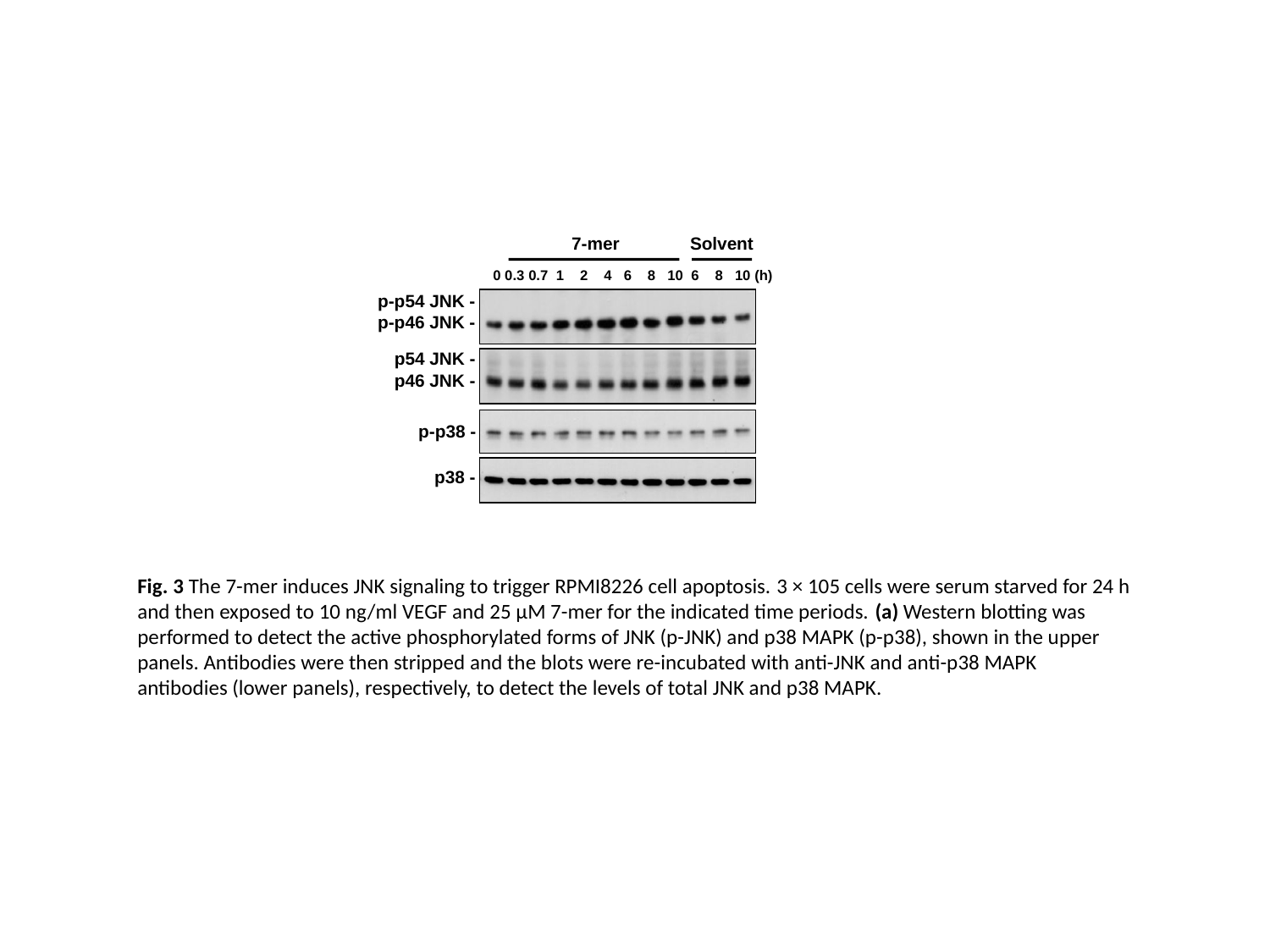

7-mer
Solvent
0 0.3 0.7 1 2 4 6 8 10 6 8 10 (h)
p-p54 JNK -
p-p46 JNK -
p54 JNK -
p46 JNK -
p-p38 -
p38 -
Fig. 3 The 7-mer induces JNK signaling to trigger RPMI8226 cell apoptosis. 3 × 10^5^ cells were serum starved for 24 h and then exposed to 10 ng/ml VEGF and 25 µM 7-mer for the indicated time periods. (a) Western blotting was performed to detect the active phosphorylated forms of JNK (p-JNK) and p38 MAPK (p-p38), shown in the upper panels. Antibodies were then stripped and the blots were re-incubated with anti-JNK and anti-p38 MAPK antibodies (lower panels), respectively, to detect the levels of total JNK and p38 MAPK.

## Slide 2
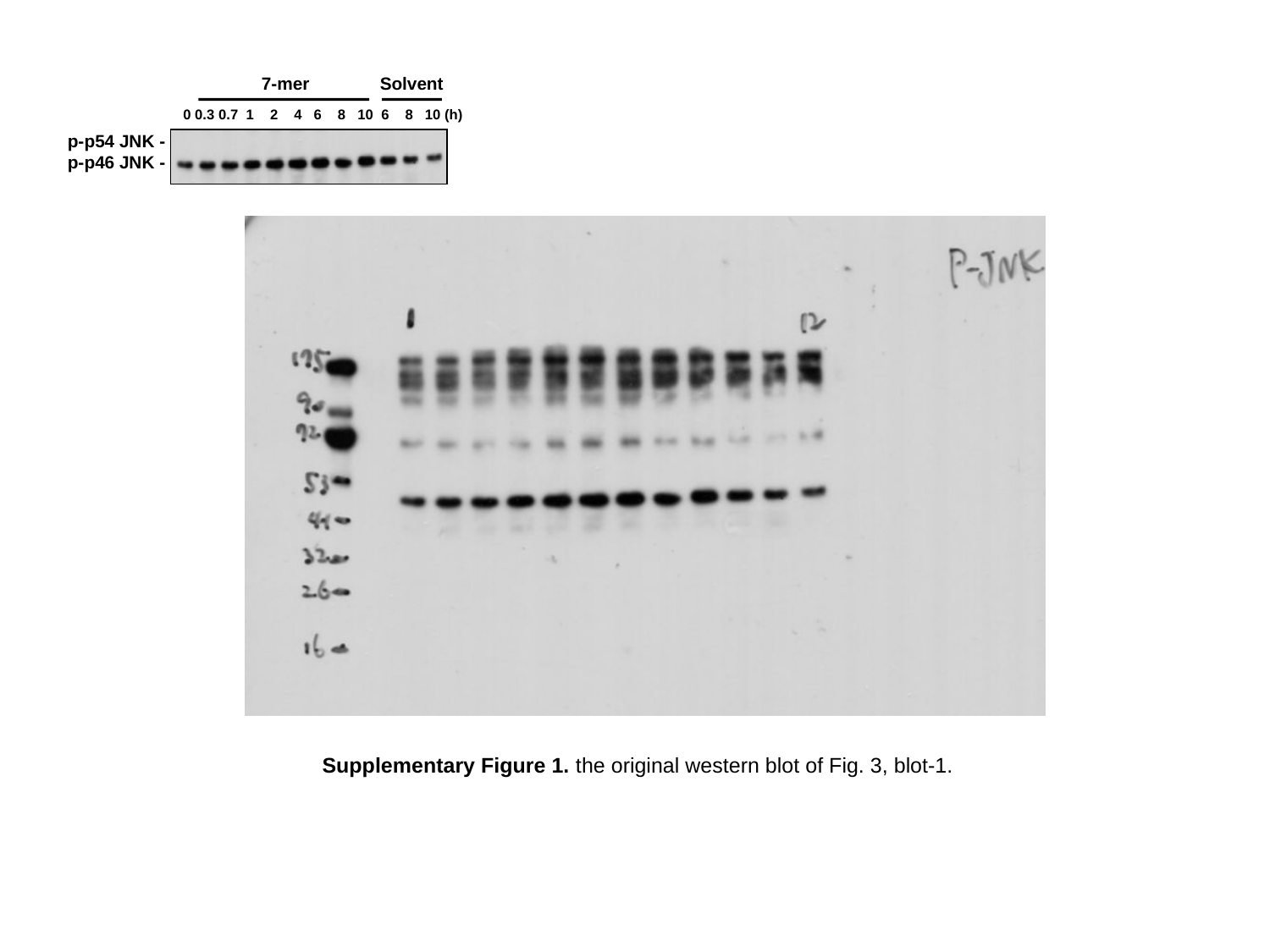

7-mer
Solvent
0 0.3 0.7 1 2 4 6 8 10 6 8 10 (h)
p-p54 JNK -
p-p46 JNK -
 Supplementary Figure 1. the original western blot of Fig. 3, blot-1.

## Slide 3
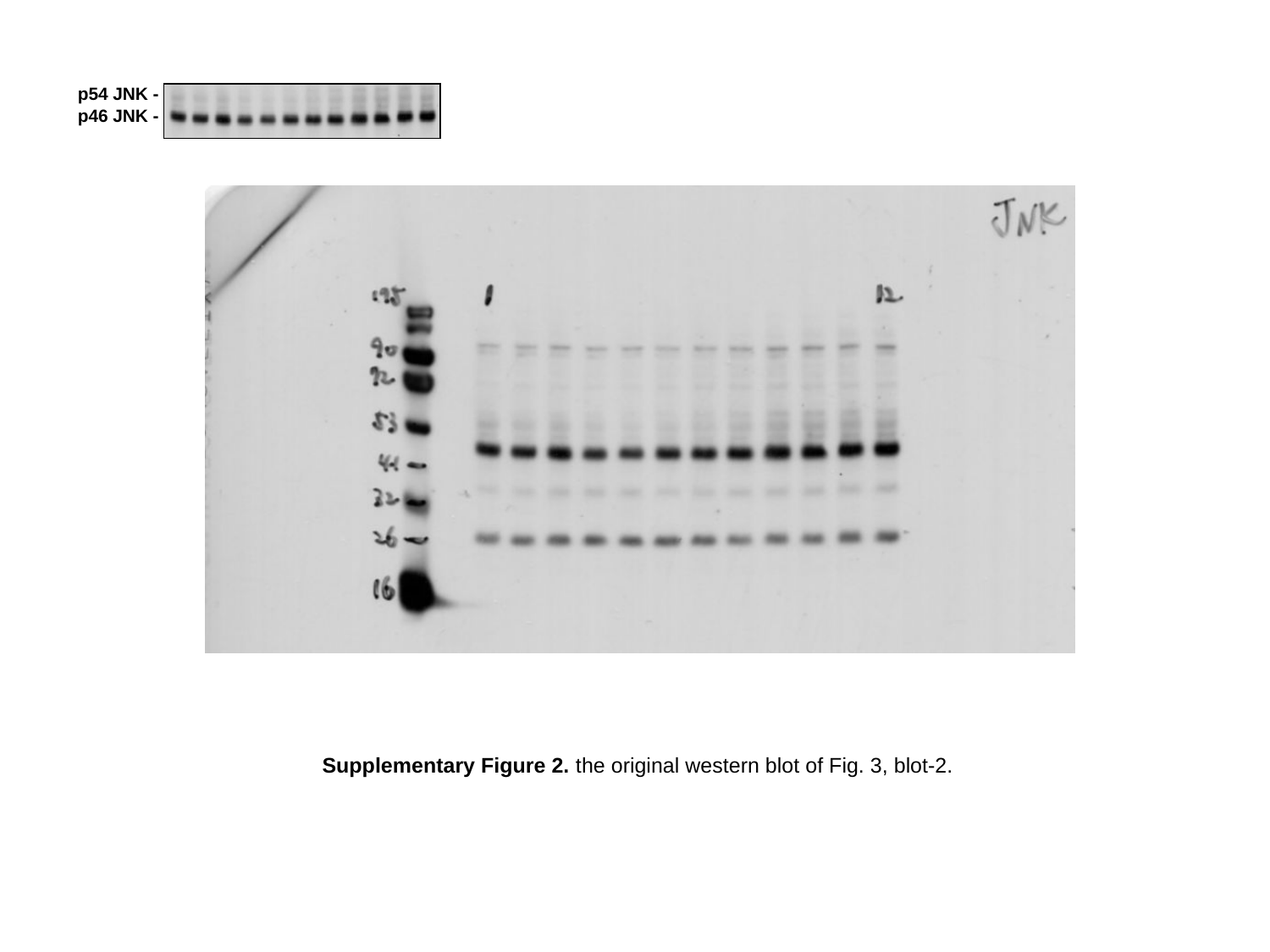

p54 JNK -
p46 JNK -
 Supplementary Figure 2. the original western blot of Fig. 3, blot-2.

## Slide 4
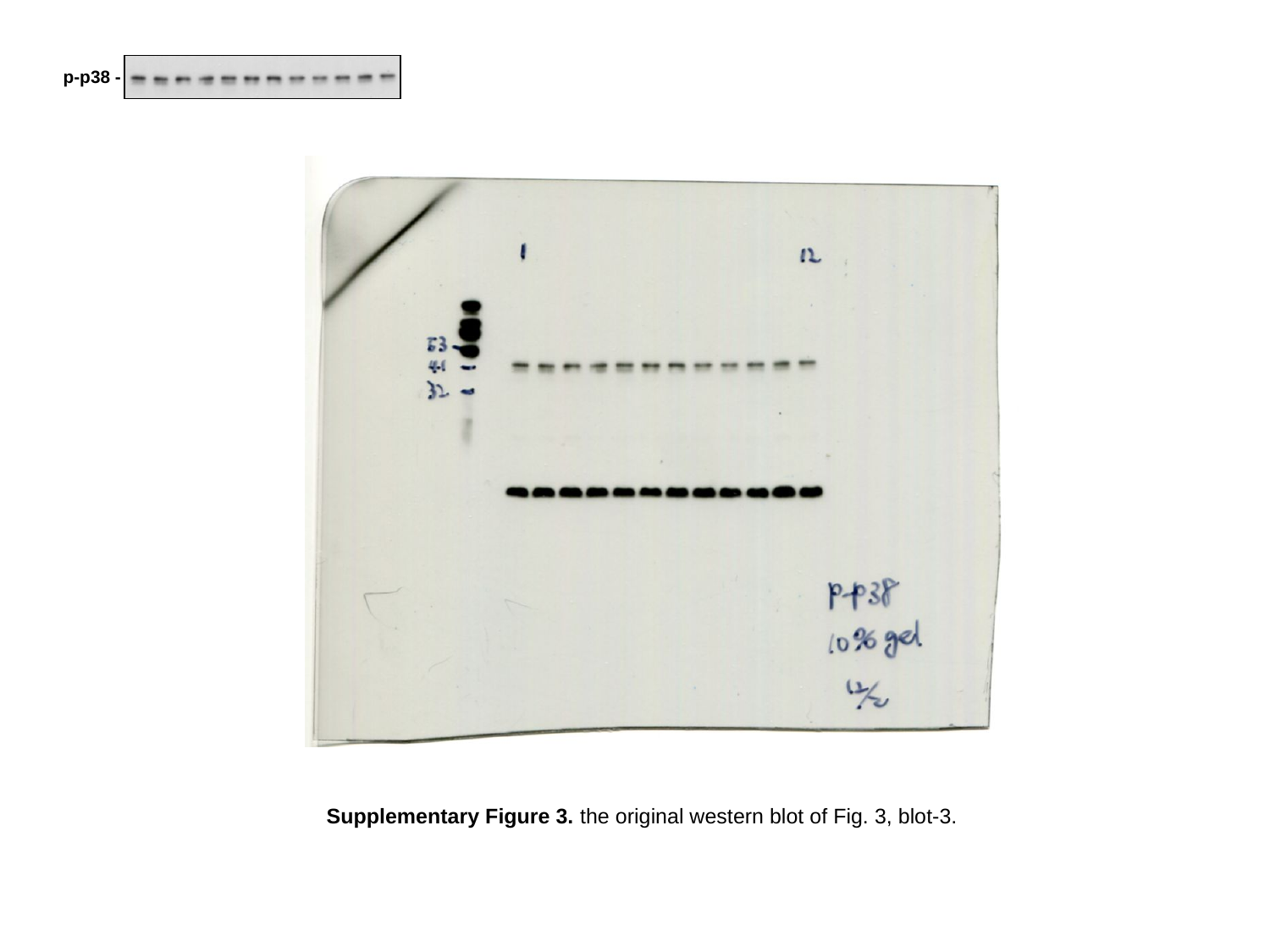

p-p38 -
Supplementary Figure 3. the original western blot of Fig. 3, blot-3.

## Slide 5
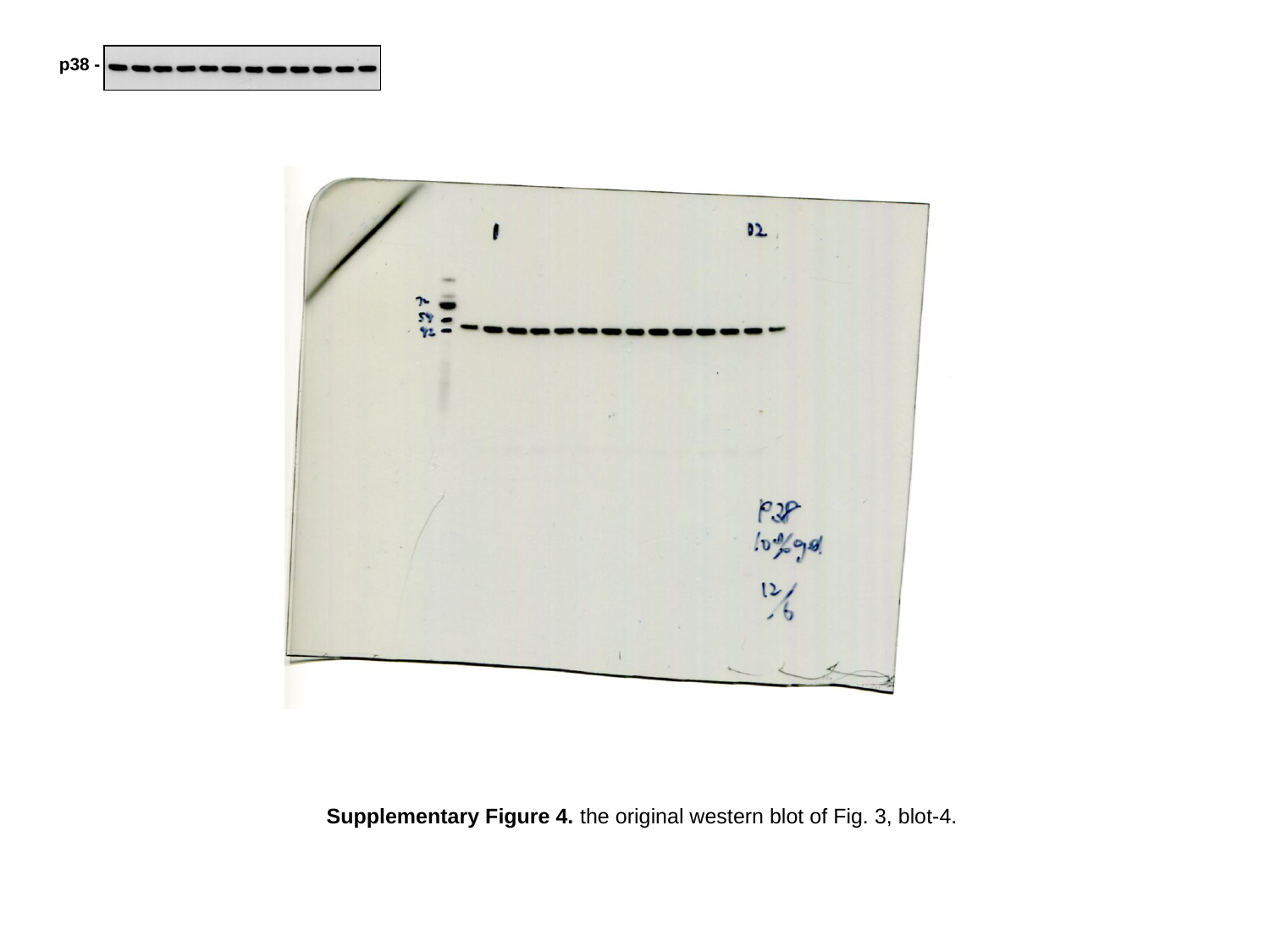

p38 -
Supplementary Figure 4. the original western blot of Fig. 3, blot-4.

## Slide 6
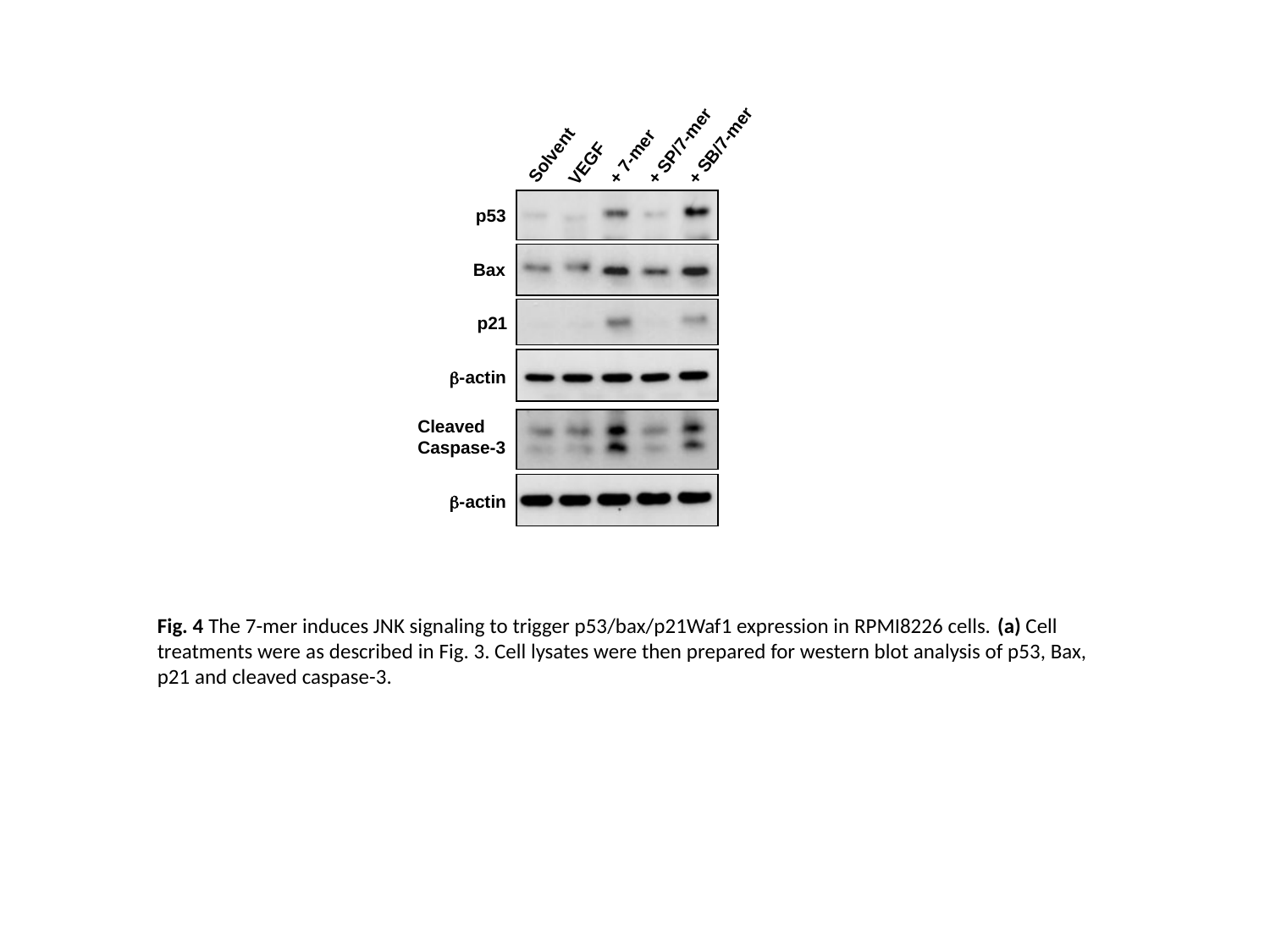

+ SB/7-mer
+ SP/7-mer
Solvent
+ 7-mer
VEGF
p53
Bax
p21
β-actin
Cleaved
Caspase-3
β-actin
Fig. 4 The 7-mer induces JNK signaling to trigger p53/bax/p21Waf1 expression in RPMI8226 cells. (a) Cell treatments were as described in Fig. 3. Cell lysates were then prepared for western blot analysis of p53, Bax, p21 and cleaved caspase-3.

## Slide 7
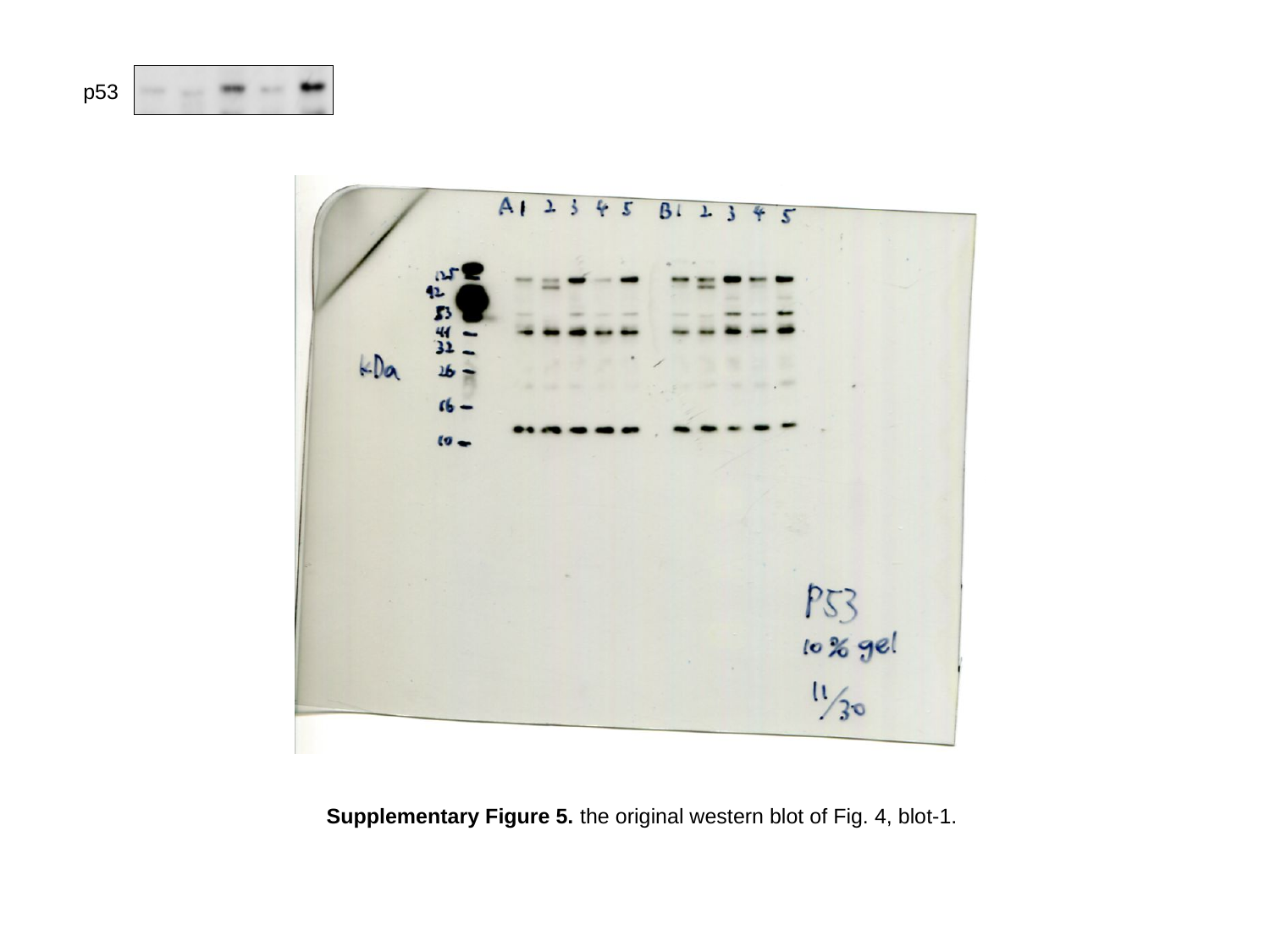

p53
Supplementary Figure 5. the original western blot of Fig. 4, blot-1.

## Slide 8
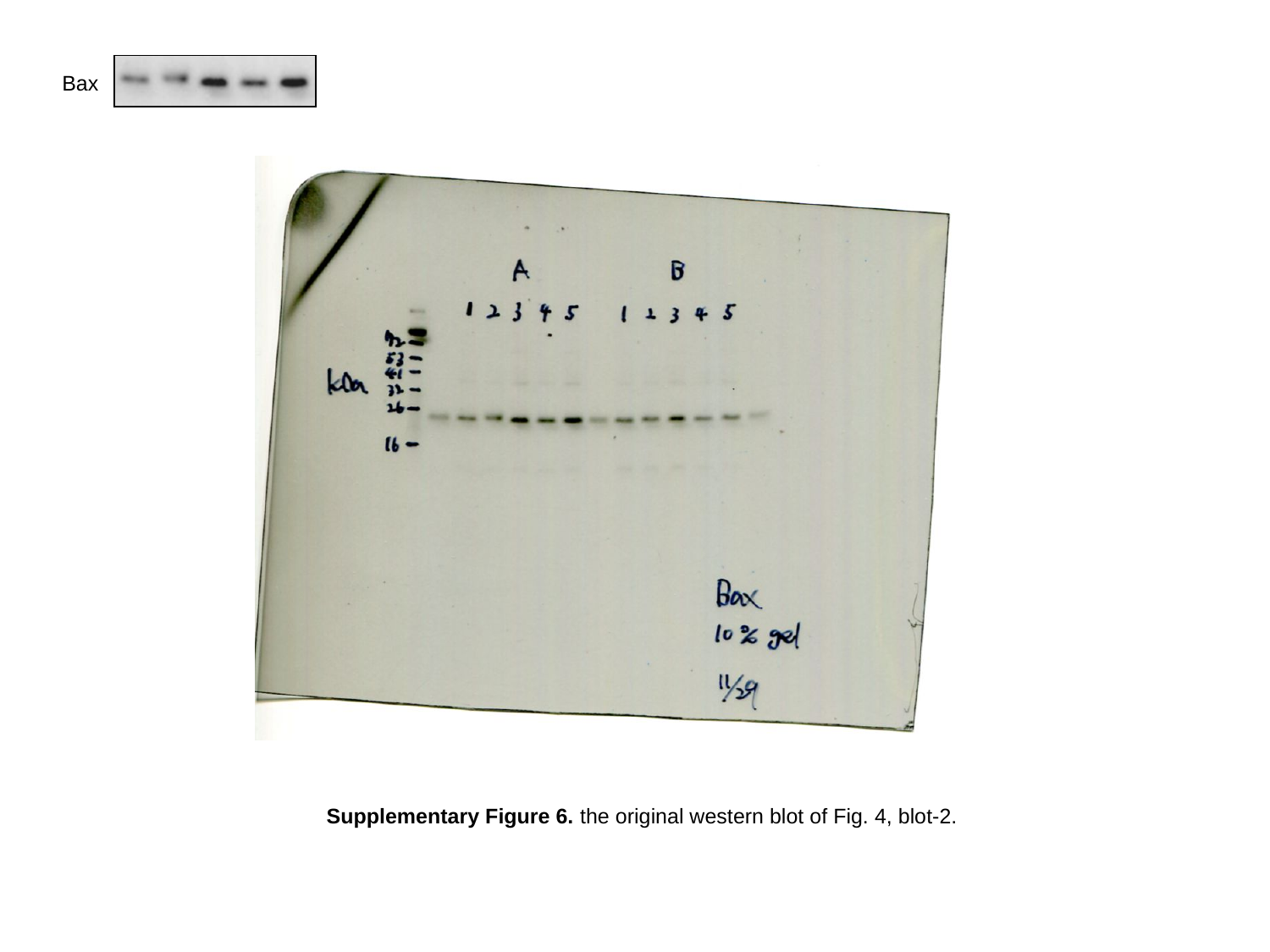

Bax
Supplementary Figure 6. the original western blot of Fig. 4, blot-2.

## Slide 9
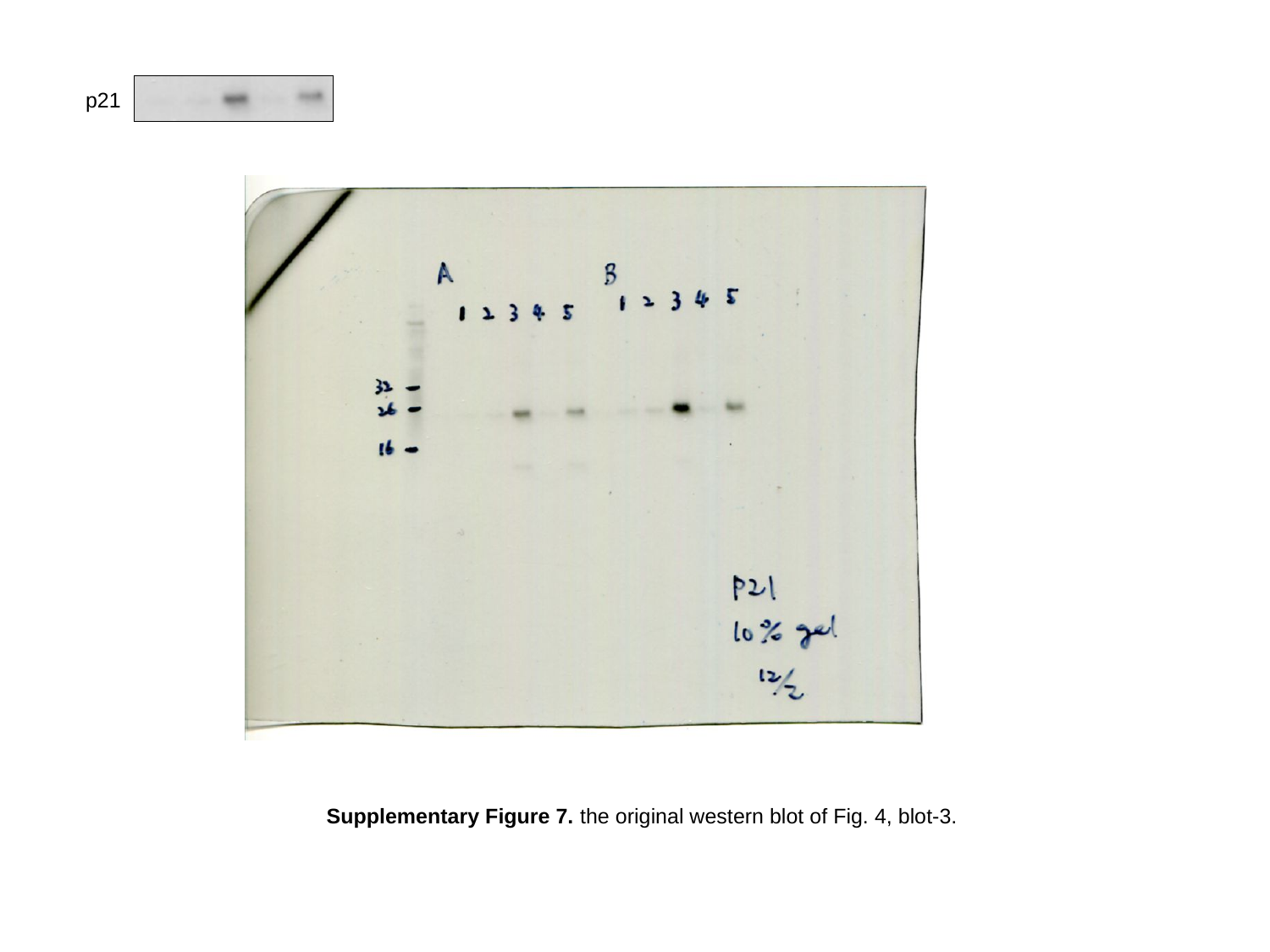

p21
Supplementary Figure 7. the original western blot of Fig. 4, blot-3.

## Slide 10
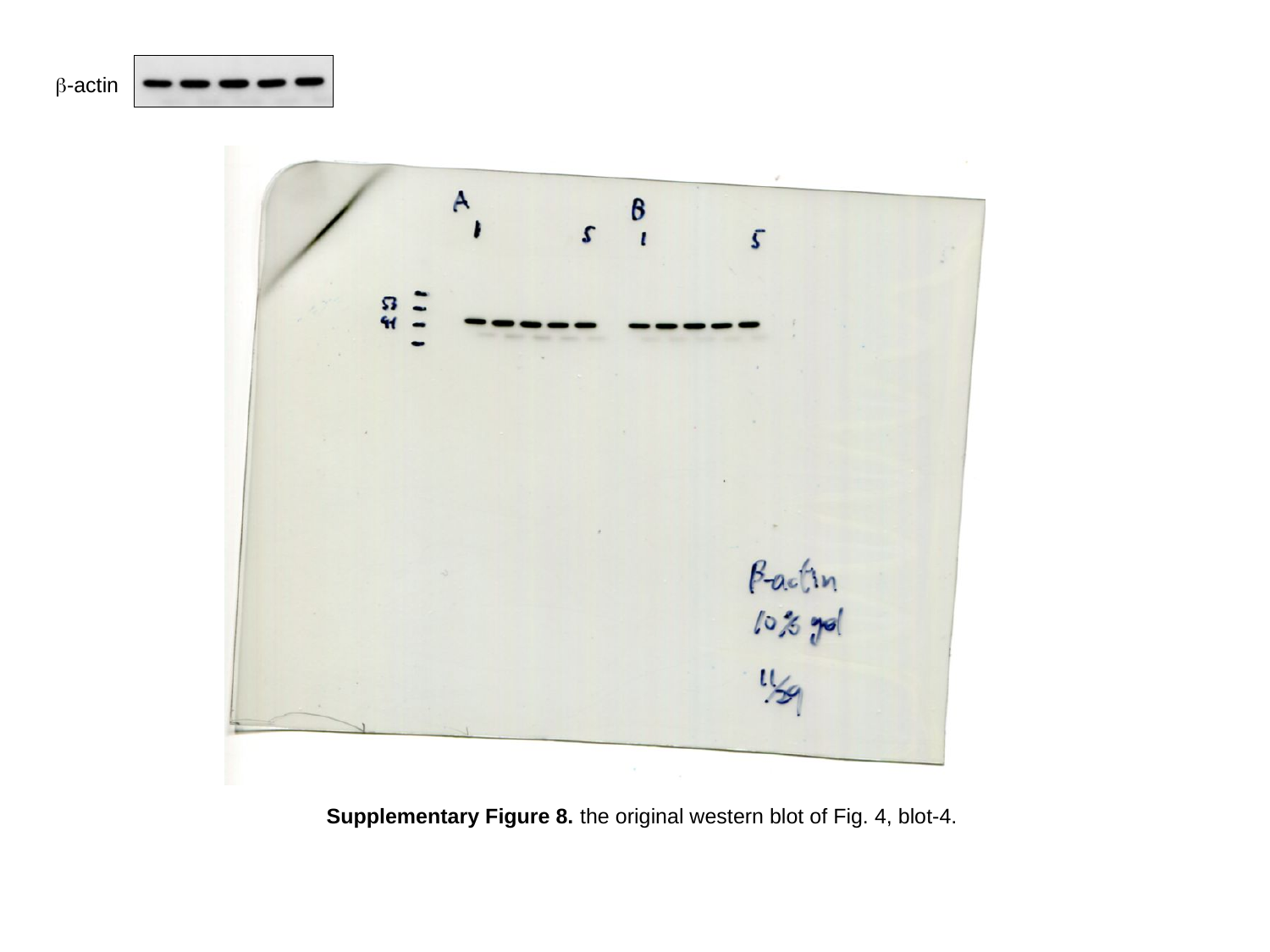

β-actin
Supplementary Figure 8. the original western blot of Fig. 4, blot-4.

## Slide 11
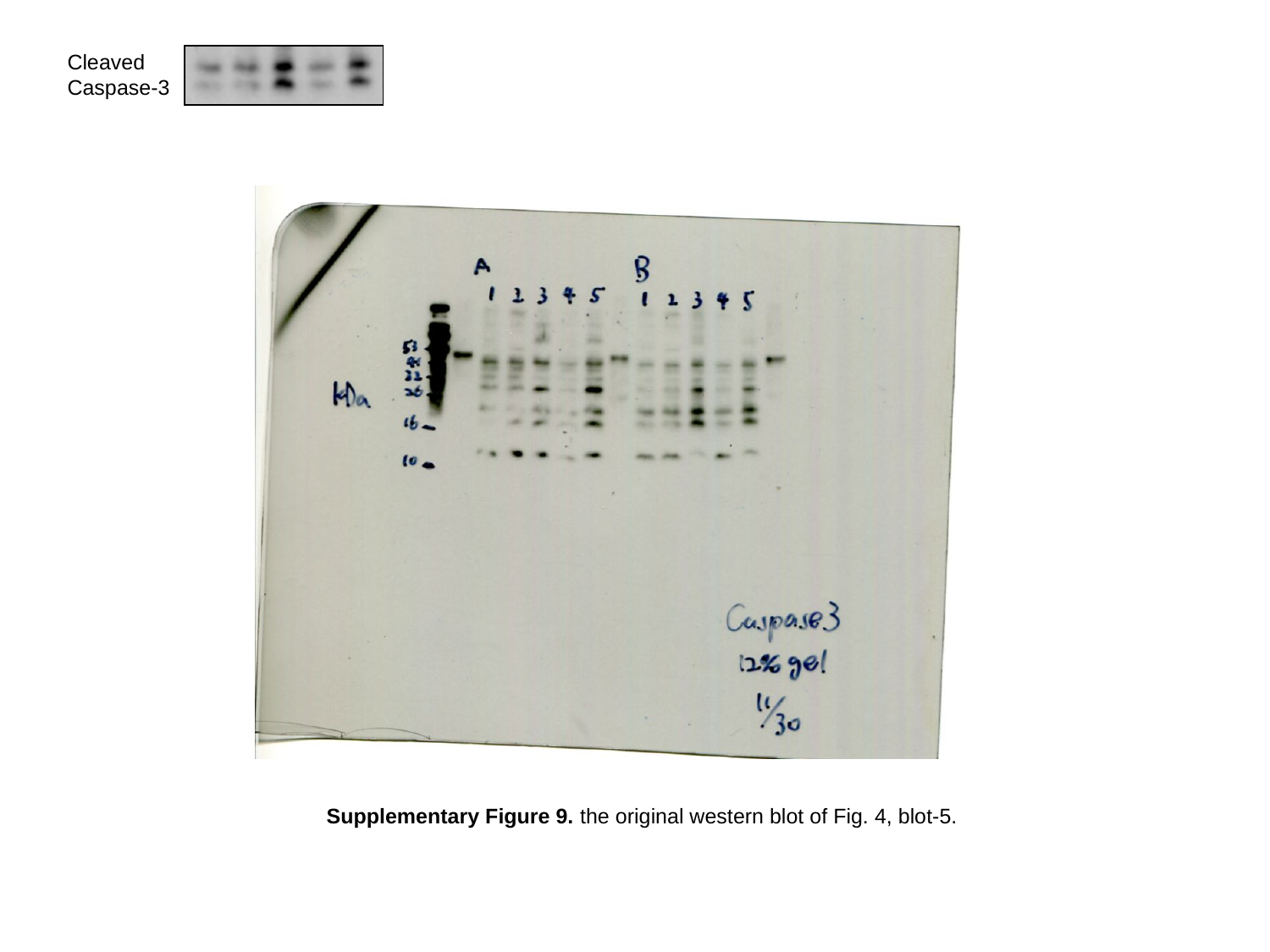

Cleaved
Caspase-3
Supplementary Figure 9. the original western blot of Fig. 4, blot-5.

## Slide 12
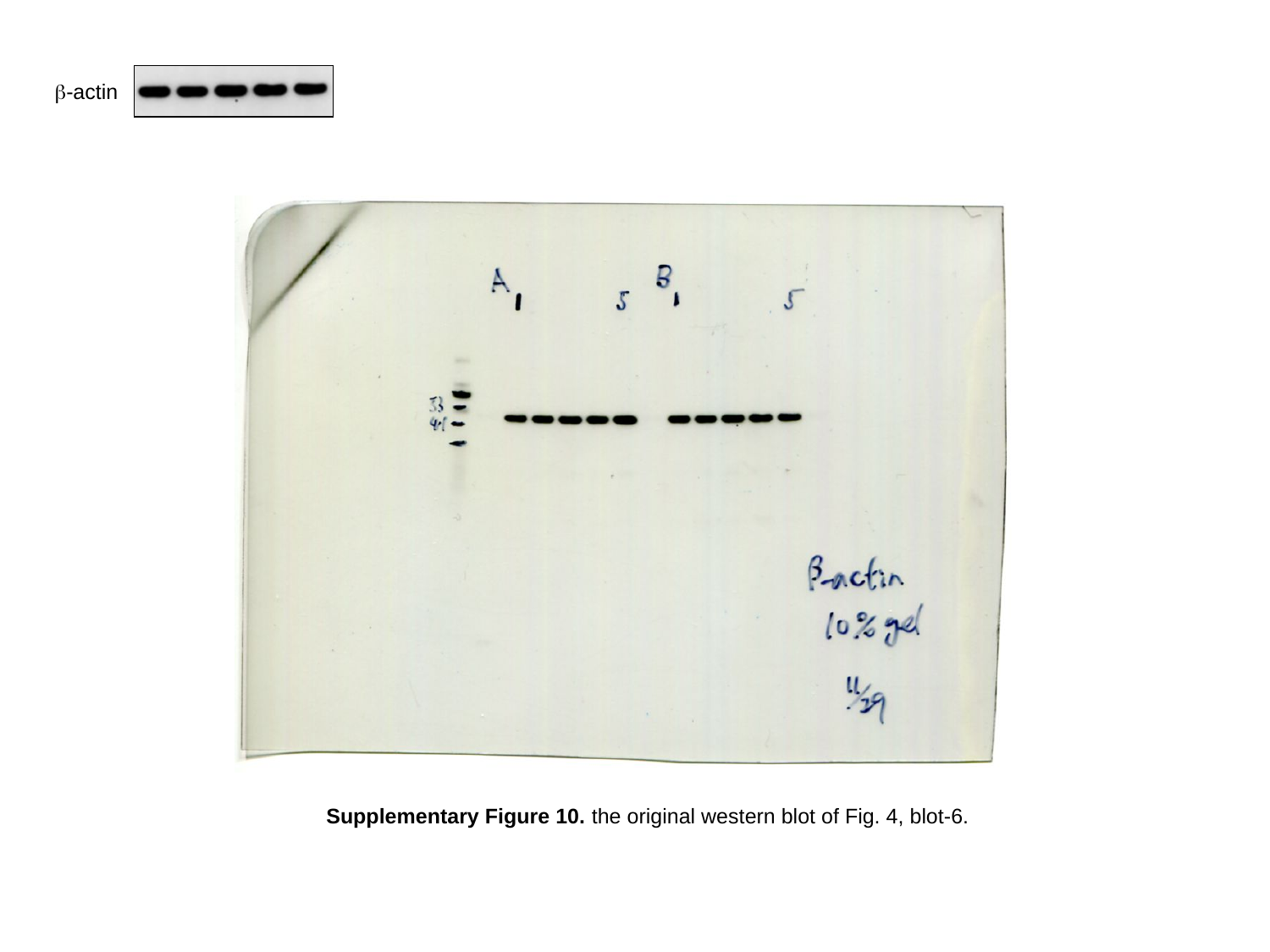

β-actin
Supplementary Figure 10. the original western blot of Fig. 4, blot-6.
